# Perception of orofacial appearance among laypersons with diverse social and demographic status

**DOI:** 10.1371/journal.pone.0239232

**Published:** 2020-09-17

**Authors:** Mohammed Nasser Alhajj, Zaihan Ariffin, Asja Celebić, Abdulaziz A. Alkheraif, Abdullah G. Amran, Ibrahim A. Ismail

**Affiliations:** 1 Department of Prosthodontics, Faculty of Dentistry, Thamar University, Dhamar, Yemen; 2 Prosthodontics Unit, School of Dental Sciences, Heath Campus, Universiti Sains Malaysia, Kelantan, Malaysia; 3 Department of Removable Prosthodontics, Faculty of Dentistry, University of Zagreb, Zagreb, Croatia; 4 Dental Biomaterials Research Chair, Dental Health Department, College of Applied Medical Sciences, King Saud University, Riyadh, Kingdom of Saudi Arabia; 5 Department of Periodontics, Faculty of Dentistry, Thamar University, Dhamar, Yemen; 6 Department of Oral Rehabilitation, Faculty of Dentistry, University of Khartoum, Khartoum, Sudan; Danube Private University, AUSTRIA

## Abstract

**Background:**

The perception of dentofacial esthetics differs considerably between patients and dentists. Patient’s expectations regarding his/her esthetics are important and should be assessed ahead of any planning of esthetic treatment. The aim of this study was to explore the differences in perception of orofacial appearance by laypersons with different social and demographic status.

**Materials and methods:**

Self-administered questionnaires were distributed to 400 subjects. The questionnaire comprised three parts; the first part addressed the demographic variables. The second part queried about dental prosthesis, orthognathic or plastic surgery, and/or ongoing or previous orthodontic treatment. The third part included the Arabic version of the-8-item Orofacial Esthetic Scale (OES-Ar) whose responses were scored in the 5-point Likert scale. These scores were compared by different grouping factors (age, gender, marital status, and education) using non-parametric Mann-Whitney U and Kruskal Willis tests with 95% confidence interval (α > 0.05).

**Results:**

A total of 268 questionnaires were eligible for analysis, representing 67% response rate. The satisfaction with facial profile appearance was the highest (4.0±1.1) followed by facial appearance (3.9±1.1), while the color of teeth was the least satisfying item (3.1±1.3). No significant differences were found between age groups for the mean summary score as well as for each item independently. No significant difference was found between both sexes except for the last item “overall impression”. Married subjects rated one item (alignment of teeth) better than their counterparts. Positive perception of orofacial appearance increased significantly with the increase of education level, the perception of the oral health status, and the perception of the general health status.

**Conclusion:**

Good oral health and/or high education level are significant determinants of more positive perception of orofacial esthetic appearance. Patients with these characteristics might be more concerned about their orofacial appearance, and this should be taken into consideration before planning any esthetic restorative dental treatment.

## Introduction

Face attractiveness is a multifactorial entity including face symmetry, nose, eyes, and dental appearance, which has been considered as the most effective feature [1, 2]. Dental appearance includes color, size, shape, and position of teeth, plus gingival display and upper lip position [3–7]. Hence, untreated carious teeth, unaesthetic restorations, and the missing of any of the anterior teeth can usually worsen the perception of dental appearance [8, 9].

Esthetic replacement or restoration has many advantages for those who had lost their teeth, particularly the anterior ones. Besides restoring functional needs, it can also improve the self-confidence and self-esteem of a patient [10, 11]. Moreover, it has been reported that proper esthetic restoration can improve the quality of life [12, 13]. Therefore, the appropriate size, shape, and color of the anterior teeth are essential in restorative dentistry.

On the other hand, the perception of dentofacial esthetics differs considerably between patients and dentists [14–17]. Patient’s expectations regarding his/her esthetics are important and should be assessed before any planning for esthetic treatment as it will be reflected directly on the patient’s feelings toward the outcome of the treatment. It is a crucial measure for the satisfaction of dental treatment [8, 18]. Some ambiguities in dental literature still exist regarding factors that influence the individual perception of dental appearance. Some researchers suggested that self-perception declined with age and that older patients have fewer concerns about the esthetics of their teeth and face [19, 20]. Other researchers, in contrast, reported that age had no significant effects on the perception of dental esthetics [8, 21].

Perception of dental and facial esthetic has been investigated using different tools; however, no study has conducted to assess the association between social and demographic factors on the perception of orofacial appearance using the orofacial esthetic scale (OES) questionnaire. The aim of this study was to explore whether there are any differences in perception of orofacial appearance, using the Arabic version of the OES questionnaire, among laypersons having different social and demographic status. The null hypothesis states no associations between different social and demographic statuses and the subject’s perception of orofacial appearance.

## Materials and methods

The study was of descriptive cross-sectional design. Subjects were consecutively recruited from the Prosthodontics and the Conservative Dentistry Departments at Faculty of Dentistry, Thamar University, Yemen. The self-administered questionnaires were distributed in two ways, either printed form or as an electronic form. The questionnaire consisted of three sections; the first section was related to social and demographic variables (age, gender, marital status, and education level), and self-rating of their oral and general health status. In this section, the education level was categorized into three levels (low educated, including illiterate and primary, moderate educated including preparatory and secondary, and high educated, including university or above). The self-rated oral and general health status were assessed using the globally well-known one question “How do you rate your oral health status?” and “How do you rate your general health status?” the responses of these questions were categorized into a 3-point Likert scale.

The second section of the questionnaire included items related to the presence or absence of dental prosthesis (implant, removable and/or fixed dental prosthesis), missing anterior teeth, orthognathic or plastic surgery, and/or ongoing or previous orthodontic treatment. The last section of the questionnaire represented the Arabic version of the Orofacial Esthetic Scale (OES-Ar) [22], which consisted of 8 items for the rating of orofacial appearance. Responses were scored by the 5-point Likert scale ranging from 1 “Very unsatisfied” to 5 “Very Satisfied”. Inclusion criteria were: 18 years or older, anterior or all teeth present (subjects with missing anterior teeth were not included), and no physical or mental disability. All subjects who answered “yes” in any question of the second section were excluded from the study. All subjects were asked to give their informed consent, and the Ethical Approval was obtained from the Research Ethical Committee, Faculty of Dentistry, Thamar University (Ref: 2019004) ahead of conducting the study.

Descriptive statistics included frequencies and proportions (for qualitative data), and means and standard deviations (summary score for quantitative data). Based on the distribution of the quantitative data, Mann-Whitney U and Kruskal Willis non-parametric tests, as appropriate, were used to compare participants’ responses to the OES-Ar and other study variables. The regression analysis was conducted to explore the determinants of the perception of the orofacial appearance. All statistical tests were performed using IBM SPSS Statistics for Windows, Version 25.0 (Armonk, NY: IBM Corp) with a 95% confidence interval and α < 0.05.

## Results

A total of 400 questionnaires were distributed. Out of the returned questionnaires (n = 350), 268 questionnaires were eligible to be included in the analysis representing 67% of the participating subjects. More than half of subjects (52.6%) were females, within the age between 21 and 30 years (66.4%), single (54.1%), and with a high education level (61.6%). More than 80% of subjects rated their general health as good, very good, or excellent. Similarly, about 70% of subjects rated their oral health as good, very good, or excellent. In general, no significant difference was found between the different age groups regarding the perception of orofacial esthetics (summary score). Also, no significant differences were found between both genders and between single and married subjects. However, significant differences were found between subgroups in relation to education level, self-rated oral health, and self-rated general health status (Table 1).

**Table 1:** Number, percentage, and mean ± SD of responses to OES-Ar by study variables.

		N (%)	Mean±SD	*P*
**Age groups**	< 20 yrs	22 (8.2)	3.5±1.0	***0*.*622***^b^
	21–30 yrs	178 (66.4)	3.7±0.9	
	> 30 yrs	68 (25.4)	3.5±1.0	
**Gender**	Male	127 (47.4)	3.7±0.9	***0*.*185***^a^
	Female	141 (52.6)	3.5±1.0	
**Marital status**	Single	145 (54.1)	3.7±0.9	***0*.*178***^a^
	Married	123 (45.9)	3.5±1.0	
**Education level**	Low	33 (12.3)	2.7±1.1	***0*.*000***^b^
	Medium	70 (26.1)	3.7±0.9	
	High	165 (61.6)	3.8±0.8	
**Perceived GH**	Poor/Fair	25 (9.3)	2.8±1.0	***0*.*000***^b^
	Good	69 (25.7)	3.3±1.0	
	Very good/Excellent	174 (64.9)	3.8±0.9	
**Perceived OH**	Poor/Fair	77 (28.7)	2.9±1.0	***0*.*000***^b^
	Good	91 (34.0)	3.7±0.7	
	Very good/Excellent	100 (37.3)	4.1±0.7	

^a^Mann-Whitney U test was used;

^b^Kruskal Willis test was used;

N: Number; SD: Standard deviation; GH: General health; OH: Oral health;

P< 0.05 is considered significant.

**Fig 1:**
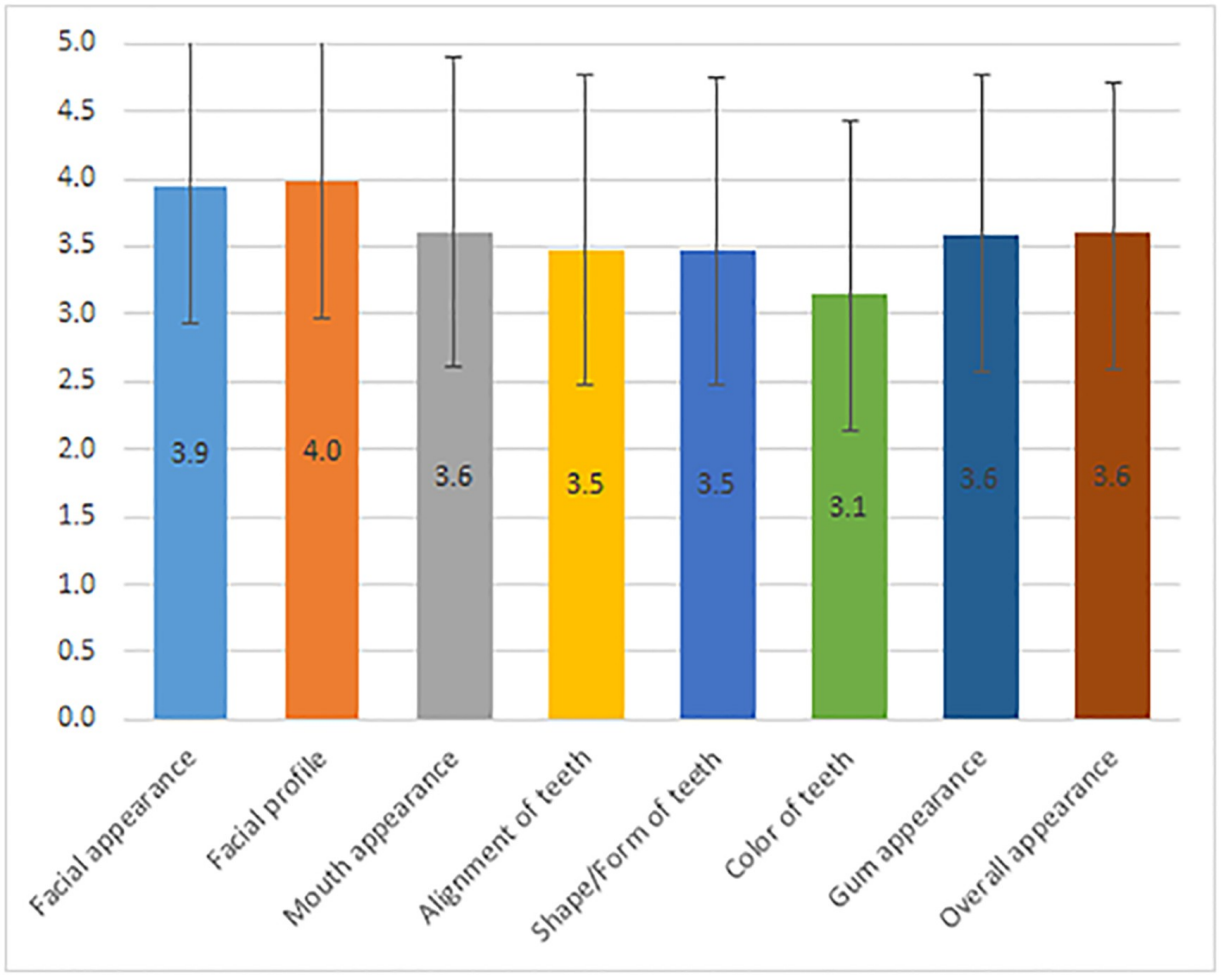
Means and SD of the 8 items of the OES-Ar for all sample.

Regarding self-perceived orofacial esthetics, satisfaction with the facial profile appearance received the highest scores (4.0±1.1), followed by the facial appearance (frontal view) (3.9 ± 1.1); the color of teeth was the least satisfying item (3.1±1.3; Fig 1). Table 2 represents the number of responses according to the questionnaire items. The color of teeth was the most unsatisfied item as scored by 37 (13.8%) participants, while the facial profile appearance was the most satisfied item as scored by 115 (42.9) participants.

**Table 2:** Distribution of responses scales according to the OES-Ar items.

	Very unsatisfied	Unsatisfied	Average	Satisfied	Very satisfied
Facial appearance	14 (5.2)	10 (3.7)	67 (25.0)	65 (24.3)	112 (41.8)
Facial profile	13 (4.9)	14 (5.2)	55 (20.5)	71 (26.5)	115 (42.9)
Mouth appearance	24 (9.0)	30 (11.2)	65 (24.3)	59 (22.0)	90 (33.6)
Alignment of teeth	27 (10.1)	37 (13.8)	58 (21.6)	75 (28.0)	71 (26.5)
Shape/Form of teeth	25 (9.3)	39 (14.6)	58 (21.6)	77 (28.7)	69 (25.7)
Color of teeth	37 (13.8)	48 (17.9)	68 (25.4)	69 (25.7)	46 (17.2)
Gum appearance	14 (5.2)	41 (15.3)	62 (23.1)	78 (29.1)	73 (27.2)
Overall appearance	15 (5.6)	28 (10.4)	70 (26.1)	93 (34.7)	62 (23.1)

No significant differences were found between both genders (P> 0.05) for all items except for the last item [overall impression (P = 0.04)]; where males rated their overall impression better than females (3.8±0.9 and 3.4±1.3, respectively). Single subjects rated their orofacial appearance better than married subjects for all items. However, these differences were statistically significant only for the color of teeth and overall impression (P = 0.04 each; Table 3).

**Table 3:** Responses to OES-Ar questionnaire according to gender and marital status.

	Gender			Marital status		
	Male	Female	*P*^a^	Single	Married	*P*^a^
Facial appearance	4.1 ± 1.0	3.8 ± 1.2	***0*.*21***	4.0 ± 1.1	3.9 ± 1.2	***0*.*76***
Facial profile	4.1 ± 1.0	3.9 ± 1.2	***0*.*26***	4.0 ± 1.1	3.9 ± 1.2	***0*.*58***
Mouth appearance	3.7 ± 1.2	3.5 ± 1.3	***0*.*35***	3.7 ± 1.2	3.4 ± 1.4	***0*.*09***
Alignment of teeth	3.4 ± 1.2	3.5 ± 1.3	***0*.*60***	3.5 ± 1.2	3.5 ± 1.3	***0*.*85***
Shape/Form of teeth	3.6 ± 1.1	3.3 ± 1.4	***0*.*14***	3.6 ± 1.2	3.3 ± 1.4	***0*.*07***
Color of teeth	3.2 ± 1.3	3.1 ± 1.3	***0*.*30***	3.3 ± 1.2	3.0 ± 1.4	***0*.*04***
Gum appearance	3.7 ± 1.1	3.5 ± 1.3	***0*.*27***	3.7 ± 1.1	3.5 ± 1.3	***0*.*36***
Overall appearance	3.8 ± 0.9	3.4 ± 1.3	***0*.*04***	3.8 ± 1.0	3.4 ± 1.2	***0*.*04***

^a^Mann-Whitney U test was used; P< 0.05 is considered significant.

Although younger subjects ≤ 30 years rated their orofacial esthetic better than subjects in age > 30 years, the differences were not statistically significant (*P*< 0.05) for all items. Positive perception of orofacial appearance increased significantly for all items with the increase of education level (*P*< 0.05); Table 4). Subjects who better rated their oral and general health status were also significantly more satisfied with their orofacial appearance (*P*< 0.05; Table 5).

**Table 4:** Responses to OES-Ar questionnaire according to age and education level.

	Age groups				Education level			
	< 20 yrs	21–30 yrs	> 30 yrs	*P*^b^	Low	Medium	High	*P*^b^
Facial appearance	3.6±1.4	4.0±1.1	4.0±1.1	***0*.*521***	3.2±1.4	4.0±1.1	4.1±1.0	***0*.*001***
Facial profile	3.9±1.2	4.0±1.1	3.9±1.1	***0*.*398***	3.1±1.4	4.1±1.1	4.1±1.0	***0*.*000***
Mouth appearance	3.6±1.3	3.7±1.3	3.4±1.4	***0*.*331***	2.8±1.5	3.6±1.2	3.8±1.2	***0*.*001***
Alignment of teeth	3.2±1.4	3.5±1.3	3.5±1.3	***0*.*683***	2.9±1.5	3.6±1.3	3.5±1.2	***0*.*049***
Shape/Form of teeth	3.2±1.2	3.6±1.3	3.3±1.3	***0*.*187***	2.3±1.3	3.4±1.3	3.7±1.1	***0*.*000***
Color of teeth	3.3±1.3	3.2±1.2	2.9±1.4	***0*.*310***	2.2±1.2	3.2±1.4	3.3±1.2	***0*.*000***
Gum appearance	3.8±1.4	3.6±1.1	3.5±1.3	***0*.*423***	2.8±1.4	3.6±1.2	3.7±1.1	***0*.*001***
Overall appearance	3.5±1.2	3.7±1.1	3.5±1.2	***0*.*485***	2.4±1.3	3.6±1.2	3.8±0.9	***0*.*000***

^b^Kruskal Willis test was used; P< 0.05 is considered significant.

**Table 5:** Responses to OES-Ar questionnaire according to self-rated oral health and general health status.

	Self-rated general health				Self-rated oral health			
	Poor	Good	Excellent	*P*^b^	Poor	Good	Excellent	*P*^b^
Facial appearance	2.8±1.2	3.6±1.3	4.2±0.9	***0*.*000***	3.2±1.4	4.0±0.9	4.4±0.8	***0*.*000***
Facial profile	2.9±1.3	3.6±1.3	4.3±0.9	***0*.*000***	3.2±1.4	4.1±0.9	4.5±0.8	***0*.*000***
Mouth appearance	3.1±1.1	3.4±1.4	3.8±1.3	***0*.*009***	2.8±1.4	3.7±1.0	4.2±1.1	***0*.*000***
Alignment of teeth	2.8±1.3	3.3±1.3	3.6±1.3	***0*.*015***	2.9±1.4	3.4±1.2	3.9±1.1	***0*.*000***
Shape/Form of teeth	2.3±1.3	3.2±1.3	3.7±1.1	***0*.*000***	2.6±1.4	3.5±1.1	4.1±1.0	***0*.*000***
Color of teeth	2.7±1.3	2.9±1.3	3.3±1.3	***0*.*017***	2.4±1.3	3.2±1.2	3.7±1.1	***0*.*000***
Gum appearance	3.0±1.4	3.4±1.2	3.7±1.1	***0*.*016***	3.0±1.3	3.5±1.1	4.1±1.0	***0*.*000***
Overall appearance	2.8±1.1	3.3±1.2	3.8±1.0	***0*.*000***	2.8±1.3	3.8±0.8	4.1±0.9	***0*.*000***

^b^Kruskal Willis test was used; P< 0.05 is considered significant.

Significant positive Spearman correlation coefficients were found between the OES-Ar summary score and the oral health score (*r* = 0.524; *P*< 0.001), as well as between the OES-Ar summary score and the general health score (*r* = 0.317; *P*< 0.001). The regression analysis (Table 6) revealed that the self-rated oral health [B = 0.20 (CI_95%_: 0.03, 0.38); *P* = 0.025] and the education level [B = 0.54 (CI_95%_: 0.39, 0.68); *P*< 0.001] were the only significant determinants of the orofacial esthetics (Model Summary: R^2^ = 0.294, Adj. R^2^ = 0.278; ANOVA < 0.001). It can be clearly noticed from the regression model that the perception of orofacial appearance would decrease with increasing age, but this relationship was not significant (*P* = 0.344).

**Table 6:** Regression analysis for the determinants affecting the perception of orofacial appearance.

	Unstandardized Coefficients		95% CI for B		*P*
	B	Std. Error	Lower	Upper	
(Constant)	1.82	0.43	0.96	2.67	***0*.*000***
Age groups	-0.10	0.11	-0.31	0.11	***0*.*344***
Gender	0.05	0.11	-0.17	0.27	***0*.*660***
Marital status	0.16	0.12	-0.09	0.40	***0*.*210***
Education level	0.20	0.09	0.03	0.38	***0*.*025***
Perceived GH	0.09	0.09	-0.09	0.27	***0*.*302***
Perceived OH	0.54	0.07	0.39	0.68	***0*.*000***

R^2^: 0.294; Adj. R^2^: 0.278; Model fit ANOVA: < 0.001;

CI: Confidence interval.

P< 0.05 is considered significant.

## Discussion

The OES questionnaire is a new tool established by Larsson et al. [23] to evaluate the perception of the individual’s orofacial esthetic. It has been translated into different languages, and its psychometric properties have been tested among different populations [22, 24–29]. The perception of esthetic can be greatly different among different populations with different sociodemographic characteristics. To the best of our knowledge, this is the first study using the OES-Ar questionnaire to explore the perception of orofacial appearance among adults of different sociodemographic levels in Yemen. The OES-Ar questionnaire used the 1–5 Likert scale for rating, as recommended in the Croatian and the Albanian version of the OES [24, 25].

The null hypothesis was partially rejected in our study as there was a significant effect of self-rated oral health and education level on the perception of orofacial appearance, while no significant effect was observed for the other factors. It has been reported that missing anterior teeth had a significant negative effect on the perception of esthetic [30, 31]. In contrast, orthodontic treatment or orthognathic/plastic surgery had a significant positive effect on esthetic perception [32]. We excluded subjects with these features or treatments to avoid any confounding factors that might affect their esthetic perception. In the current study, the questionnaires were distributed either in hard or electronic form. Neophytou et al. [33] reported no impact of administration mode on the assessment of orofacial appearance using the OES.

In general, our observations revealed that facial profile appearance had the highest self-rated scores, followed by the frontal facial appearance, while the color of teeth received the worst ratings. However, these observations had no significant association with age, gender, and marital status of the participants. The color of teeth is a sensitive and substantial component of smile appearance and attractiveness [34–37]. Although in our study, younger individuals perceived their orofacial appearance better than their counterparts, no significant difference was found between age groups. Tin-Oo et al. [8] found no significant effect of age on perception of a dental appearance, while some other studies [19, 38] found significant effects of age. The variations of the results might be due to differences in the scales which had been used. We found out that the perception of the esthetics between both genders was significantly different for the overall esthetic impression. This overall result was in favor of males who reported better orofacial appearance. In general, females are more concerned about the esthetics and, therefore, tend to rate their dental appearance lower than males [39–42].

Single individuals rated their orofacial appearance better than married ones. This might be due to the fact that married individuals had more responsibilities and more priorities in their life than taking care of their appearance. Individuals with high education levels rated their orofacial appearance better than those with low or none education. This result is in agreement with some previous studies [41–43]. Individuals with higher academic achievement seem to be more aware of and more concerned about their oral health and orofacial appearance and probably have better hygiene, less carious lesions, which might be responsible for their better scores.

It is well documented that the satisfaction of dental aesthetics plays an essential role in the perception of oral health and oral health-related quality of life [13, 22, 44–47]. Moreover, the relationship between oral and general health status has been reported [46, 48–50]. The perception of the oral and facial complex from the patient’s point of view is of utmost importance for the success, as well as the satisfaction of dental treatment [39, 51, 52]. Social variation among individuals is another factor that should be considered.

Though the present study clearly showed some differences in perception of orofacial appearance among individuals with social disparities, being most of the participants within the age < 30 years and with high education level should be acknowledged as limitations, and the results could not be generalized for the whole population. Another shortcoming of the study could be the use of single questions to measure the oral and general health although the OES can be used as a stand-alone instrument when a detailed assessment of esthetic concerns is desired [23]. The small sample size and the low response rate is another limitation for interpreting the results. Therefore, the present study could be a starting point for further community-based studies with larger sample size and more socioeconomic and demographic factors may be necessary.

## Conclusion

Within the limitations of this study, it can be concluded that good oral health (self-rated) and/or high education level are independent determinants of more positive perception of orofacial esthetic appearance. Patients with these characteristics might be more concerned about their orofacial appearance, and this should be taken into consideration before planning any esthetic restorative dental treatment.

## Supporting information

S1 Data (XLSX)

S1 File (DOCX)

## Abbreviations

Ar: Arabic
OES: Orofacial Esthetic Scale
