# Supplementary material for: Perception of orofacial appearance among laypersons with diverse social and demographic status
Source: PLoS One. 2020 Sep 17;15(9):e0239232. doi: 10.1371/journal.pone.0239232 (PMC7498058; doi:10.1371/journal.pone.0239232)
Supplement: S1 File — (DOCX) [file pone.0239232.s002.docx]

| **استبــيـــــان حول التقييم الذاتي لمظهر الوجه، الفم، و الأسنان و علاقته بالخصائص الإجتماعية** | | | | | | | | |
| --- | --- | --- | --- | --- | --- | --- | --- | --- |
|  |  | | |  |  | |  | |
| نرجو تكرمكم مشكورين بالإجابة على الأسئلة المدونة أدناه والمتعلقة بالتقييم الذاتي لمظهر الوجه و الفم و الأسنان. يحتوي الاستبيان على عدة أسئلة تحدد مدى قناعتك بمظهر الوجه و الفم و الأسنان لديك، بالإضافة إلى بعض الأسئلة المتعلقة بذلك. | | | | | | | | |
| (المعلومات التي سيتم جمعها هي لغرض البحث العلمي و لن يطلع عليها أحد). | | | | | | | | |
| **التعـلـيمـــات:** |  | | |  |  | |  | |
| 1- التأكد من قراءة السؤال جيداً قبل الإجابة. | | | | |  | |  | |
| 2- الإجابة على الاستبيان لمرة واحدة فقط. | | | | |  | |  | |
| 3- يمكنك استخدام المرآة إذا لزم الأمر. | | | | |  | |  | |
| 4- إجابة واحدة فقط لكل سؤال (ضع إشارة √ على الاجابة المتعلقة بك). | | | | | | |  | |
|  |  | | |  |  | |  | |
| **العـــمر** |  | | |  |  | |  | |
| أقل من 20 | من 20 إلى 30 | | | أكثر من 30 |  | |  | |
| **الجنـــس** |  | | |  |  | |  | |
| ذكــــر | أنثـــى | | |  |  | |  | |
| **الحـالة الاجتمـاعيـة** |  | | |  |  | |  | |
| عازب/ة | متزوج/ة | | |  |  | |  | |
| **المستـوى التعـليـمي** | | | |  |  | |  | |
| لا يقرأ ولا يكتب / إبتدائي | إعدادي / ثانوي | | | جامعة فما فوق |  | |  | |
| **ما هو تقييـمك لصحتك العـامة؟** | | | |  |  | |  | |
| ضعيفة / مقبـولة | جيـدة | | | جيـدة جداً / ممتـازة | | |  | |
| **ما هو تقييـمك لصحتك الفمـويـة؟** | | | |  |  | |  | |
| ضعيفة / مقبـولة | جيـدة | | | جيـدة جداً / ممتـازة | | |  | |
| **هل يوجد لديك أسنان مقلوعة في المنطقة الأمامية من الفم؟** | | | | | | | | |
| نعم | | لا |  | | |  | |  |
| **هل يوجد لديك تركيبات صناعية في المنطقة الأمامية من الفم؟** | | | | | | | | |
| نعم | لا | | |  |  | |  | |
| **هل لديك معالجة تقويم للأسنان حالياً أو قمت بعمل معالجة تقويم في وقت سابق؟** | | | | | | | | |
| نعـم | لا | | |  |  | |  | |
| **هل خضعت لعملية جراحية أو تجميلية لمنطقة الوجه، الفم أو الفكين؟** | | | | | | | | |
| نعـم | لا | | |  |  | |  | |
| **ما هو شعورك نحو مظـهر وجـهك؟** | | | |  |  | |  | |
| غير مقتنع جداً | غير مقتنع | | | وسط | مقتنع | | مقتنع جداً | |
| **ما هو شعورك نحو المظهر الجانبي لوجهك؟** | | | | |  | |  | |
| غير مقتنع جداً | غير مقتنع | | | وسط | مقتنع | | مقتنع جداً | |
| **كيف تشعر حول مظهر الثلث الأسفل لوجهك؟** | | | | |  | |  | |
| غير مقتنع جداً | غير مقتنع | | | وسط | مقتنع | | مقتنع جداً | |
| **ما هو شعورك نحو مظهر فمـــك (الإبتسامة، الشفاه و الأسنان الظاهرة)؟** | | | | | | | | |
| غير مقتنع جداً | غير مقتنع | | | وسط | مقتنع | | مقتنع جداً | |
| **ما هو شعورك نحو مظهر اصطفاف/صفوف أسنانك؟** | | | | | | |  | |
| غير مقتنع جداً | غير مقتنع | | | وسط | مقتنع | | مقتنع جداً | |
| **ما هو شعورك نحو حول شكـل أسنانك؟** | | | |  |  | |  | |
| غير مقتنع جداً | غير مقتنع | | | وسط | مقتنع | | مقتنع جداً | |
| **ما هو شعورك نحو لــون أسنانك؟** | | | |  |  | |  | |
| غير مقتنع جداً | غير مقتنع | | | وسط | مقتنع | | مقتنع جداً | |
| **ما هو شعورك نحو مظـهر لثتك؟** | | | |  |  | |  | |
| غير مقتنع جداً | غير مقتنع | | | وسط | مقتنع | | مقتنع جداً | |
| **إجمالاً، ما هو شعورك نحو مظهر وجهك، فمك و أسنانك؟** | | | | | | |  | |
| غير مقتنع جداً | غير مقتنع | | | وسط | مقتنع | | مقتنع جداً | |
